# Supplementary material for: Transcriptome analysis in whole blood reveals increased microbial diversity in schizophrenia
Source: Transl Psychiatry. 2018 May 10;8:96. doi: 10.1038/s41398-018-0107-9 (PMC5943399; doi:10.1038/s41398-018-0107-9)
Supplement: Supplementary file 1 — Supplementary Materials [file 41398_2018_107_MOESM1_ESM.docx]

Supplemental Materials

**Supplementary Methods**

**Sample recruitment**

Schizophrenia, bipolar patients and control subjects included in this study were recruited at the University Medical Center Utrecht, The Netherlands. Detailed medical and psychiatric histories were collected, and only patients with a DSM-IV diagnosis of schizophrenia or bipolar disorder were included as cases; controls were neurologically healthy individuals, free of any recorded psychiatric history (1-3). ALS patients were recruited from ALS clinics at UCLA and UCSF.

**Sample preparation and sequencing**

Whole blood was collected in PAXgene Blood RNA tubes and total RNA was isolated using the PAXgene extraction kit (Qiagen). For all samples, RIN values were obtained using Agilent’s RNA 6000 Nano kit and 2100 Bioanalyzer, and measures for RNA concentration were obtained using the Quant-iT RiboGreen RNA Assay Kit. Sequencing was performed by UCLA Sequencing Core using the Illumina Hiseq 2000 platform.

RNASeq of B-lymphoblast cell lines

HapMap B-lymphoblast cell lines (n=3) were used for the negative control experiment (see Supplemental Results section **validation and potential contamination**) were cultured up to 5 days. All cell lines were grown in RPMI 1640 (Sigma-Aldrich) supplemented with 15% fetal bovine serum (Fisher Scientific) and 2mM L-glutamine (Fisher Scientific)  at 37C and 5% (vol/vol) CO2 in a humidified incubator. Cell pellets were lysed with Buffer RLT and RNA was extracted using the RNeasy Mini Kit (Qiagen). Just as our discovery samples, we used the TruSeq RNA v2 library preparation, including Ribo-Zero Gold rRNA depletion. Samples were collected from a trio (father, mother, offspring) in duplicate. We obtained 144.6 million 2x69bp paired-end reads (Rapid run).

Whole Blood Exome Sequencing

We used exome sequencing as an independent technology to validate the detected microbial composition in our RNA-Seq cohort (see Supplemental Results section **validation and potential contamination**). Whole blood was collected in EDTA tubes and the extraction performed using the Kleargene XL blood DNA extraction kit. DNAseq libraries were prepared using Illumina’s TruSeq protocol, using the TruSeq Exome enrichment kit.

**Sequence Analysis**

TopHat v. 2.0.12 was used to filter read pairs and singleton reads mapped to the human genome or transcriptome (default parameters, Ensembl GRCh37 transcriptome and Ensembl hg19 build). TopHat2 was supplied with a set of known transcripts (as a GTF formatted file, Ensembl GRCh37) using –G option. After sub-sampling to 100,000 reads, we filtered out low-quality and low-complexity reads; that is, reads with at least 75% of their base pairs having quality lower then 30 (FASTX, <http://hannonlab.cshl.edu/fastx_toolkit/>) and reads with sequences of consecutive repetitive nucleotides (SEQCLEAN, <http://sourceforge.net/projects/seqclean/>). Next, the remaining reads were realigned to the reference human genome and transcriptome (Ensembl GRCh37 transcripome and Ensembl hg19 build) using the Megablast aligner (BLAST+ 2.2.30, edit distance 6) (4) to filter out any remaining potentially human reads. We prepared the index from each reference sequence using makembindex from BLAST+. The following parameters were used for makembindex: iformat = blastdb. To map the reads using Megablast, we used for each reference the settings task = megablast, use_index = true, perc_identity = 94, outfmt =6, max_target_seqs =1. We consider only entirely mapped reads in further analyses.

**Beta diversity**

We calculate beta diversity per each combination of the samples resulting in a matrix of all pair-wise sample dissimilarities. Bray-Curtis beta diversity index is measured taxonomically as $\boldsymbol{1}-\frac{\boldsymbol{2}\boldsymbol{J}}{\boldsymbol{A}+\boldsymbol{B}}$, where J is the sum of the lesser values for the shared taxa, A and B are the sum of the total values for all taxa for each sample, respectively. Total diversity of the groups is estimated as a function of the total number of taxa (gamma diversity). We use gamma diversity to estimate diversity of the group as well as total diversity of the study.

**Statistical analysis of microbiome diversity**

To test for differences in alpha diversity between disease groups, we fit the following analysis of covariance (ancova) model

$$alpha\_norm \sim Sex + Age + Technical covariates + Disease status$$

, where *Alpha_norm* = alpha values after inverse normal transformation and *Age* = Individual’s age at blood draw. Technical covariates include: *RIN, Batch (Plate_number)*, *Concentration*, and *Flow cell lane*, wher*e RIN* = RNA integrity value, a measure for RNA quality and *Concentration* = RNA concentration prior to normalization at the genotyping core.

The effect of disease status was estimated by first regressing out the effects of the included covariates. Adjustment for pairwise comparisons for all possible disease status pairs (6 comparisons) was performed using Bonferroni correction for multiple testing.

To determine the relative effect size of alpha diversity on schizophrenia status, we fit the following logistic regression model:

$$SCZ \sim Sex + Age + Technical covariates + alpha\_norm$$

Where *SCZ* is a binary variable, which is coded as true if the sample belongs to the SCZ cohort.

Variation explained is by *alpha_norm* was measured by the reduction in R^2^, comparing the full logistic regression model versus a reduced model with *alpha_norm* removed.

We separately tested for differences in alpha by sex or age directly within each group by correlating normalized alpha values with sex/age, using Spearman rank correlation.

To assess difference in Beta diversity we fit a similar model as above, now correcting for sex, age and technical covariates for each individual:

$$beta\_norm \sim Sex1 + Sex2+ Age1 + Age2 + Technical covariates1 + Technical covariates2 + Group$$

where *beta_norm* = beta values for each pair of individuals after inverse normal transformation, and *Group* contains set SCZ_SCZ (both individuals from SCZ), SCZ_Control (one SCZ, one control), Control_Control (both controls).

Adjustment for pairwise comparisons for all possible disease status pairs (three comparisons) is performed using Bonferroni correction for multiple testing. We also determined a possible effect of alpha diversity on the above model by adding normalized values of alpha as a covariate to the model. In addition to our ANCOVA analysis, we performed a Permutational Multivariate Analysis of Variance (PERMANOVA) using the beta value distance matrix based on 1,000 permutations (using the R function *adonis* from the *vegan* package).

**Reference-free microbiome analysis**

We complemented the reference-based taxonomic analysis with a reference independent analysis. We used EMDeBruijn (<https://github.com/dkoslicki/EMDeBruijn>), a reference-free approach that is capable of quantifying differences in microbiome composition between the samples. EMDeBruijn compresses the k-mer counts of two given samples onto de Bruijn graphs and then measures the minimal cost of transforming one of these graphs into the other (in terms of how many k-mers moved how far). This direct comparison of samples allows one to circumvent the many issues involved with selecting a phylogenetic classification algorithm, choosing which training database to use and deciding how to compare two classifications.

Other reference-free comparison metrics have previously been used (such as treating k-mer frequencies as vectors in $\mathbb{R}^{n}$ and then using the Euclidean distance, Jensen-Shannon divergence, Kullback-Liebler divergence, cosine similarity, etc.). However, treating k-mer frequencies as vectors in $\mathbb{R}^{n}$ ignores the dependencies induced by the amount of overlap between two given k-mers. Instead of Euclidean space, EMDeBruijn considers k-mer frequencies as existing on an underlying de Bruijn graph, a structure that naturally takes into consideration such overlap-induced dependencies.

Fixing a k-mer size, we first formed the undirected de Bruijn graph, with vertices given by k-mers, and an edge between two k-mers if the first (or last) k-1 nucleotides of one k-mer overlaps with the last (or first) k-1 nucleotides of the other k-mer. Let $d(\cdot,\cdot)$ represent the resulting graph distance. Then, given two metagenomic samples, $S_{1}$and $S_{2}$, we let the frequencies of k-mer be given by ${freq}_{k}(S_{1})$ and ${freq}_{k}(S_{2})$ respectively. These frequencies are thought of as weights on the vertices of the de Bruijn graph. Next, to represent the transformation of one set of weights into the other, we used the term flow (or coupling) which is any real-valued matrix $\gamma$ with rows and columns indexed by k-mers, such that the row sums equals ${freq}_{k}(S_{1})$ and the column sums equal ${freq}_{k}(S_{2})$. A flow represents how much weight was moved where. There are infinitely many flows possible, but we chose the most efficient flow, which is defined to be the one that minimizes the total cost (in terms of weight times distance). This leads to the definition of the EMDeBruijn metric ${EMD}_{k}\left( S_{1},S_{1} \right):$

$${EMD}_{k}\left( S_{1},S_{1} \right):= \min_{\gamma} \sum_{x,y \mathrm{kmers}} \gamma\left( x,y \right)*d(x,y)$$

Hence, the EMDeBruijn metric measures the minimal cost of transforming one sample’s k-mer frequency vector into the other sample’s k-mer frequency vector, when allowable transformations are restricted to moves along edges of the de Bruijn graph. To compute this quantity, we used the FastEMD implementation of the Earth Mover’s Distance since the graph metric $d(\cdot,\cdot)$ is naturally thresholded. We found that a good trade-off between algorithmic run-time and effectiveness of the resulting metric was to use the k-mer size of k=6.

To determine the variation explained by EMdeBruin principal components, we adopted a similar approach as described above and fit the following logistic regression model:

$$SCZ \sim Sex + Age + Technical covariates + PC1+PC2+PC3$$

where *PCi* denotes the ith EMdeBruin principal component.

**Correlation of microbial diversity with genetic risk for schizophrenia**

To determine a correlation between genetic risk for schizophrenia and alpha diversity, we compared alpha diversity to the polygenic risk score for schizophrenia. The polygenic risk score represents the cumulative genetic load of disease risk alleles and is defined as the sum of trait-associated alleles across many genetic loci, weighted by effect sizes estimated from a genome-wide association study. We based our scores on the most recent genome wide association study (5) with our samples removed (6), and used a P-value cut-off of *P* < 0.05. For a total of 32 schizophrenia cases, we had both polygenic risk score and alpha diversity measures available, and we performed a Spearman rank correlation. We obtained similar results using different P-value cutoffs to determine the polygenic risk score.

**Estimation of DNA methylation-derived cell proportions in whole blood**

DNA methylation profiles of heterogeneous tissue types reflect variability in underlying cellular composition (29, 30). Recent studies, using flow-sorted cell populations, identified CpG sites discriminatory for distinct cell populations and developed sophisticated methods to estimate blood cell proportions from DNA methylation data derived from whole blood (28-30). We used these methods to investigate a potential link between microbial diversity and the immune system.

In a control cohort of 220 individuals, blood-based genome-wide methylation data was collected using the Infinium HumanMethylation450 BeadChip. We used the epigenetic clock software (7) with normalization to estimate cell abundance measures. Briefly, this software uses Houseman’s estimation method (8, 9) to estimate monocytes, granulocytes, CD8 T, CD4 T, natural killer and B cells. In addition, it predicts abundance measures for plasmablasts (i.e., immature plasma cells), CD8.naive, CD4.naive and CD8pCD28nCD45RAn cells (i.e., differentiated CD8 T cells), all based on a penalized elastic net regression model (7, 10).

Quality control of the DNA methylation data was performed as follows**.** CpG sites with bead counts less than 5 or a detection p-value greater than 0.01 in more than 5% of samples were removed using the pfilter function in the wateRmelon package in R. In addition, samples having more than 5% of CpG sites with a detection p-value greater than 0.01 or having gender discrepancies were excluded from further analyses. Next, we removed CpG sites with probes containing known SNPs (EUR, MAF > 0.01) and probes that are cross-reactive, i.e. non-specific (11, 12). Data was background corrected using the danen function in R (wateRmelon package) and beta values were extracted for further analyses.

We investigated the relationship between microbiome diversity and the immune system as follows. From a cohort of n=220 controls for which methylation-derived cell proportions were available, we first obtained residuals for each cell-type using the following model:

$$Proportion\_cell\_type \sim Sex + Age + Beadchip + Beadchip position + dataset.$$

In addition, we used residuals from the above-described regression on alpha diversity using our full replication cohort. Using all samples with both alpha levels and methylation-based cell abundance measures available (a total of n=65), we next fitted a linear regression model with alpha diversity residuals as a response variable and all blood cell proportion residuals as independent variables. Each independent variable was analyzed as it was put in the model last to account for correlations among cell proportions. We therefore model the relationship between alpha diversity and individual cell types while adjusting for all other cell types.

**Supplementary Results**

**Validation and potential contamination**

We performed the following negative control experiment to investigate the possibility of DNA contamination introduced during RNA isolation, library preparation and sequencing steps,. We applied our ‘lost and found’ pipeline to RNA-Seq reads from six B-lymphoblast cell line (LCLs) samples. LCLs are cultured in a sterile tissue culture environment using aseptic techniques. We therefore expect these to lack any presence of microbial species. Neither PhyloSift nor MetaPhlAn detected bacterial or archaeal microorganisms in the LCLs samples (See Table S1.C). This experiment also serves as a positive control, as the only virus detected by PhyloSift is the Epstein-Barr virus, used for transfection and transformation of lymphocytes to lymphoblasts (13).

We used a more direct positive control dataset to validate the feasibility of using human RNA-Seq for detection of microbial organisms and applied the ‘lost and found’ pipeline to RNA-Seq data collected from epithelial cells infected with Chlamydiae (Humphrys et al. 2013). The authors collected data using ribo-depletion and polyA selection protocols at 1 and 24 hours post infection. PhyloSift was able to detect the Chlamydiae phylum in 100,000 reads randomly subsampled from unmapped reads*,* confirming the validity of the bioinformatics pipeline used (Table S3).

The design of experimental procedures such as blood draw and subsequent downstream lab procedures may lead to global contamination effects. In our data, there is minimal evidence that the detected microbial communities are confounded by contamination due to experimental procedures. First, all RNA samples were subjected to the same standardized RNA isolation protocols, library preparation and sequencing procedures. With the exception of Proteobacteria, which has been reported to be the most abundant phylum in whole blood (14), we observe no phylum present in all individuals, suggesting absence of a uniform contaminator due to experimental procedures applied across all samples.

Second, we collected two blood tubes per individual, of which one was randomly chosen for subsequent RNA sequencing. If skin contamination upon first blood draw occurs, due to contact with the needle, its effect will be randomly distributed across half of individuals in our cohort and should therefore not affect downstream between-group analyses.

Third, it is vital to scrutinize the potential impact of parameters that are variable between samples, such as experimenter (i.e., lab technician who extracted RNA from blood collections) (15). To investigate these potential effects, we grouped samples by various experimental variables, including sequencing run and experimenter. We observed no evidence that the detected microbial communities are confounded by contamination, which is in agreement with previously reported studies that documented at most a low background signal introduced by such variables (14) (See also Figure S1 and S2). In addition, we included all available technical covariates, such as RNA integrity number (RIN), batch, flow cell lane and RNA concentration, in our disease specific analyses.

Finally, an independent technology was used to validate the detected microbial composition in our RNA-Seq cohort. We used available blood whole exome sequence data from two individuals from the cohort (See Table S1.B). We applied the ‘lost and found’ pipeline and compared results from both technologies. Despite the use of different technologies and reagents, microbiome profiles from both sequencing procedures were found to be in close agreement. For both individuals, we were able to detect several microbial phyla, all of which were also identified using RNAseq. Conversely, RNAseq was able to detect several microbial phyla not detected using exome sequencing (Table S4). Taken together, these results confirm the validity and potential of our ‘lost and found’ pipeline.

**Comparing blood microbiome to HMP**

Our data suggest that the predominant phyla of the blood microbiome are most closely related with the known oral and gut microbiome. That is, out of eight blood microbiome phyla detected in at least 50% of HMP samples, all are found in the oral or stool samples. Among those four phyla (Spirochaete, Deferribacteres, Chlamydiae, Fusobacterium) are found in the majority of the oral or stool samples, but in less than 50% of other tissues. The Bacteroidetes phylum was present in the majority of the oral, stool and skin samples. The remaining three phyla (Actinobacteria, Firmicutes, and Proteobacteria) were present in the majority of HMP samples across all tissues. The majority of the eight phyla not confirmed by HMP have been described to be present in or on the human body. For example, Crenarchaeota, Thermotogae, Deinococcus-Thermus and Planctomycetes have been associated with the human gut microbiome (16-19) Other phyla are reported to be present in other human tissues (e.g., Thaumarchaeota, which is present in skin microbiome)

**Alpha diversity using only younger samples**

We repeated the analysis of alpha diversity using only younger samples (with Age<47, the maximum age in the schizophrenia cohort, resulting in n=107 samples), because the groups included in the schizophrenia sample had large age differences and were younger on average. Here, we obtained similar results (i.e. *ANCOVA* P < 0.007) between schizophrenia and all other groups, and we found no significant differences observed between BPD, ALS and Controls.

**Relationship between alpha and beta diversity**

To investigate whether the differences in beta diversity are driven by our primary observation of increased alpha diversity, we corrected for alpha diversity in our model. While we still observed Controls_Controls < SCZ_Controls and SCZ_SCZ< SCZ_Controls at P<0.001, the beta values of Controls_Controls, and SCZ_SCZ were no longer significantly different (Figure S2). Thus, these observations are not entirely driven by the increase in microbial diversity observed in SCZ samples.

**Relationship between alpha diversity and the number of microbial reads being detected by PhyloSift.**

While we normalized the number of unmapped reads to 100,000 for each sample, we hypothesized that the increased alpha diversity observed in schizophrenia may be due to an increased number of microbial reads being detected by PhyloSift in these samples. However, if we add number of reads detected as a covariate to our regression analysis the results remain unchanged (*ANCOVA* P < 0.005 for all groups with SCZ, and no significant differences were observed between the groups BPD, ALS and Controls). The average number of reads detected by PhyloSift are: SCZ (1049 ± 458), BPD (1375 ± 295), ALS (1289 ± 494) and Controls (1226 ± 478).

**Beta diversity and EMDeBruijn analysis in the replication sample**

For beta diversity, the pattern we observed in the replication sample slightly diverged from the results obtained from our discovery cohort: while Controls_Controls still has the lowest average beta diversity, we observed increased beta diversity in SCZ_SCZ group versus SCZ_Controls (P < 0.0001 Figure S4). One potential explanation for this discrepancy is that beta diversity in the replication sample was computed at the genus rather than phylum level, making slight mismatches between individuals more likely, and distances between samples harder to compute based on present microbial taxa. This is expected to be more likely if both samples have a large microbial diversity. In relation to this, contrary to what we observed in the discovery sample, we did not observe a correlation between EMDeBruijn distances and Beta diversity in this sample.

However, as in our discovery sample, EMDeBruijn PCs significantly correlated with principal components that were obtained from edge PCA based on the MetaPhlAn taxonomic classification (Correlation between EMDeBruijn PC1, and MetaPhlAn PC1 is P = 6.091e-06, rho = -0.32 Spearman rank correlation, see also Figure S5). Finally, as in our discovery sample, the first three EMDeBruijn principal components adjusted for covariates were significant predictors of status and together explain 7.8% of the variance.

**Supplementary Figures**


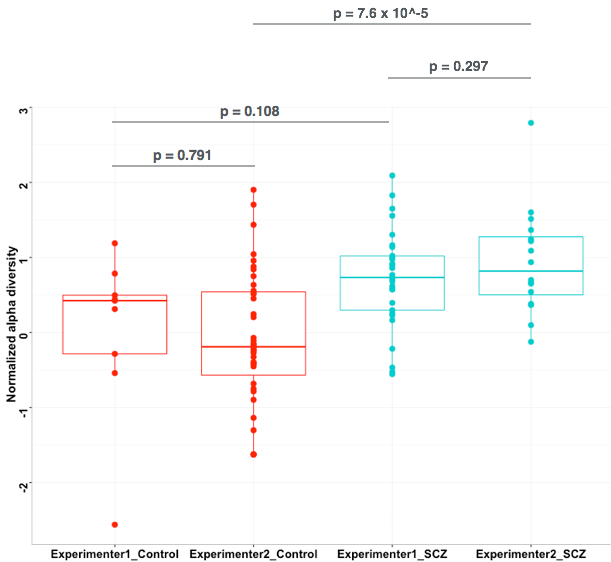


**Fig S1:** Experimenter and alpha diversity measures. We analyzed whether experimenter (lab technician) can account for differences in alpha diversity between controls and schizophrenia. We find no evidence that experimenter has an effect. We included all samples for which this information was available (sample sizes: Experimenter1_Control=9, Experimenter2_control=39, Experimenter1_SCZ=29, Experimenter2_SCZ=16). Student t-test was used to evaluate differences between groups, and p-values are reported.


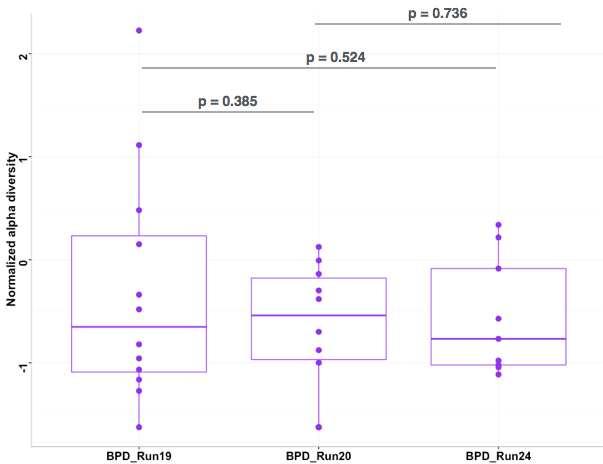


**Fig. S2:** RNA isolation run and alpha diversity measures. We assessed whether alpha diversity measures vary across isolation runs. We included only runs for which at least 8 samples were included (sample sizes: Run19=12, Run20=10, Run24=9) and found no evidence for this. Student t-test was used to evaluate differences between runs, and p-values are reported.

**Fig. S3:** Alpha diversity per sample for four subject groups (Controls, ALS, BPD, SCZ), measured using the inverse Simpson index for main taxonomic ranks (Class, Order, Family, Genus).

**Fig. S4:** Beta diversity measured using Bray-Curtis beta diversity metric across pairs of samples from the SCZ (N=48) and Controls (N=49), resulting in three subject groups: SCZ_Controls, SCZ_SCZ, Controls_Controls. Figures A shows the raw beta values. Figures B and C show beta diversity residuals after correcting for covariates (sex and age, RNA INtegrity (RIN) value, Plate, Flow cell lane and RNA concentration) (B), as well as alpha levels (C), respectively. Figures D, E and F are analogous plots for the replication study of schizophrenia cases (N=91) and controls (N=88).

**Fig. S5:** Principal component analysis of microbial reads. All samples were analyzed by dimensionality-reduction methods to estimate trends in community composition with respect to disorder status. Discovery sample: A) PCoA applied to a similarity matrix. Similarities between samples were calculated using the EMDeBruijn metric. B) Edge PCA applied to a matrix where columns correspond to edges in the reference phylogeny, rows correspond to each sample. Each entry is the difference in placed sequence probability masses on either side of that edge calculated by Guppy using the PhyloSift output. Replication sample: C) PCoA applied to a similarity matrix. Similarities between samples were calculated using the EMDeBruijn metric. D) PCoA applied to a similarity matrix. Similarities between samples were calculated using Jensen-Shannon divergence on the normalized genus level reconstructions obtained by MetaPhlAn.

**Fig. S6:** Correlation between the proportion of CD8+ CD28- CD45RA- cells and corrected alpha levels at the genus level after correcting for all other cell-count estimates and technical covariates based on 65 control samples from the replication cohort.

**Supplementary Tables**

**Table S1: Data Overview**

*[provided as pdf file]*

**Table S2: Similarities of blood microbiome profiles with other body sites**

*[provided as pdfl file]*

**Table S3: Relative abundance of the Chlamydiae phylum at 1 and 24 hours post infection**

Genomic abundances of Chlamydiae phylum for RNA-Seq samples prepared by ribo-depletion and polyA selection protocols at 1 and 24 hours post infection.  Phylogenetic classification is performed by PhyloSift for 100,000 reads randomly subsampled from unmapped reads*.*

| **time** | **polyA RNA-Seq** | **Ribo-Zero RNA-Seq** |
| --- | --- | --- |
| 1 hour | 0% | 0.1% |
| 24 hour | 24% | 0.9% |

**Table S4: Cross Technology Validation of RNA-Seq versus Exome sequencing**

| **Sample** | **Microbial Phyla** | **Exome sequencing** | **RNA-Seq** |
| --- | --- | --- | --- |
| Sample1 | FIRMICUTES | ✔ | ✔ |
| Sample1 | ACIDOBACTERIA | ✔ | ✔ |
| Sample1 | PROTEOBACTERIA | ✔ | ✔ |
| Sample1 | CYANOBACTERIA |  | ✔ |
| Sample1 | AQUIFICAE |  | ✔ |
| Sample1 | PLANCTOMYCETES |  | ✔ |
|  |  |  |  |
| Sample2 | PROTEOBACTERIA | ✔ | ✔ |
| Sample2 | FIRMICUTES | ✔ | ✔ |
| Sample2 | THERMOTOGAE |  | ✔ |
| Sample2 | CYANOBACTERIA |  | ✔ |
| Sample2 | CHLAMYDIAE |  | ✔ |
| Sample2 | AQUIFICAE |  | ✔ |
| Sample2 | VERRUCOMICROBIA |  | ✔ |

**Table S5:** **Alpha diversity.** P-values of differences in alpha diversity across group by ANCOVA including confounding factors of sex and age, and technical covariates (RNA INtegrity (RIN) value, Plate, Flow cell lane and RNA concentration) using normalized values of alpha after Bonferroni correction for multiple testing. P-values that survive correction for multiple testing are marked bold.

| **Disease Status** | **Control** | **SCZ** | **BPD** | **ALS** |
| --- | --- | --- | --- | --- |
| Control | - | **0.0032** | 1 | 1 |
| SCZ | - | - | **0.0003** | **0.0128** |
| BPD | - | - | - | 1 |
| ALS | - | - | - | - |

**Table S6: Cell count estimates and Alpha diversity** Correlation of normalized and corrected alpha diversity values with cell-count estimates after correction for all other cell-count estimates, based on 73 controls from the replication sample. P-values that survive correction for multiple testing are marked bold.

| **Cell Type** | **Method** | **t-value** | **p-value** |
| --- | --- | --- | --- |
| Monocytes | Houseman | -0.33 | 0.74 |
| Granulocytes | Houseman | -0.26 | 0.79 |
| CD8 T cells | Houseman | 0.14 | 0.89 |
| CD4 T cells | Houseman | -0.88 | 0.38 |
| B cells | Houseman | 0.41 | 0.68 |
| NK cells | Houseman | -0.5 | 0.62 |
| CD8.naive | Horvath | -1.05 | 0.3 |
| CD4.naive | Horvath | -1.5 | 0.14 |
| Plasmablast | Horvath | 0.55 | 0.58 |
| CD8pCD28nCD45RAn | Horvath | -3.58 | **0.00073** |

**Table S7: Microbial phyla prevalence across individuals and disorders**. P-values that survive correction for multiple testing are marked bold. Phyla marked with a * are present in at least 1/4^th^ of the samples in each subject group.

| **Microbial Phyla** | **Controls (Controls)** | **Amyotrophic Lateral Sclerosis (ALS)** | **Bipolar Disorder (BPD)** | **Schizophrenia (SCZ)** | **Total** | **P-value Fisher** | **P-value corrected** |
| --- | --- | --- | --- | --- | --- | --- | --- |
|  |  |  |  |  |  |  |  |
| Fusobacterium | 0 | 0 | 0 | 1 | 1 | 1.0000 | 1.0000 |
| Elusimicrobia | 1 | 1 | 0 | 1 | 3 | 1.0000 | 1.0000 |
| Acidobacteria | 2 | 0 | 0 | 1 | 3 | 0.6209 | 1.0000 |
| Tenericutes | 0 | 1 | 0 | 2 | 3 | 1.0000 | 1.0000 |
| Deinococcus-Thermus | 1 | 0 | 2 | 1 | 4 | 0.9561 | 1.0000 |
| Synergistetes | 0 | 2 | 1 | 2 | 5 | 0.9561 | 1.0000 |
| Aquificae | 1 | 0 | 0 | 4 | 5 | 0.1826 | 1.0000 |
| Nitrospirae | 3 | 0 | 1 | 2 | 6 | 0.5012 | 1.0000 |
| Spirochaetes | 4 | 0 | 1 | 1 | 6 | 0.4851 | 1.0000 |
| Chlamydiae | 1 | 2 | 1 | 4 | 8 | 0.9004 | 1.0000 |
| Chloroflexi | 2 | 2 | 1 | 4 | 9 | 0.8710 | 1.0000 |
| Verrucomicrobia | 3 | 1 | 4 | 3 | 11 | 0.8516 | 1.0000 |
| Deferribacteraceae | 3 | 0 | 2 | 9 | 14 | 0.0092 | 0.2126 |
| Thaumarchaeota | 6 | 4 | 4 | 4 | 18 | 0.9678 | 1.0000 |
| Bacteroidetes | 3 | 5 | 2 | 15 | 25 | 0.0888 | 1.0000 |
| Planctomycetes | 5 | 3 | 2 | 20 | 30 | 0.0002 | **0.0057** |
| Thermotogae | 6 | 3 | 6 | 20 | 35 | 0.0006 | **0.0141** |
| Euryarchaeota | 11 | 16 | 15 | 6 | 48 | 0.0669 | 1.0000 |
| Crenarchaeota | 18 | 16 | 17 | 4 | 55 | 0.0215 | 0.4955 |
| Actinobacteria | 15 | 10 | 12 | 19 | 56 | 0.2420 | 1.0000 |
| Cyanobacteria* | 28 | 19 | 15 | 32 | 94 | 0.0043 | 0.0991 |
| Firmicutes* | 36 | 29 | 32 | 43 | 140 | 0.0130 | 0.2988 |
| Proteobacteria* | 48 | 46 | 48 | 48 | 190 | 0.8853 | 1.0000 |

**Supplementary References**

1. Buizer-Voskamp JE, Muntjewerff JW, Genetic R, Outcome in Psychosis Consortium M, Strengman E, Sabatti C, et al. Genome-wide analysis shows increased frequency of copy number variation deletions in Dutch schizophrenia patients. Biol Psychiatry. 2011;70(7):655-62.

2. Loohuis LM, Vorstman JA, Ori AP, Staats KA, Wang T, Richards AL, et al. Genome-wide burden of deleterious coding variants increased in schizophrenia. Nat Commun. 2015;6:7501.

3. Ripke S, O'Dushlaine C, Chambert K, Moran JL, Kahler AK, Akterin S, et al. Genome-wide association analysis identifies 13 new risk loci for schizophrenia. Nat Genet. 2013;45(10):1150-9.

4. Camacho C, Coulouris G, Avagyan V, Ma N, Papadopoulos J, Bealer K, et al. BLAST+: architecture and applications. BMC bioinformatics. 2009;10:421.

5. Schizophrenia Working Group of the Psychiatric Genomics Consortium. Biological insights from 108 schizophrenia-associated genetic loci. Nature. 2014;511:421--7

.

6. Ripke S, O'Dushlaine C, Chambert K, Moran JL, Kahler AK, Akterin S, et al. Genome-wide association analysis identifies 13 new risk loci for schizophrenia. Nature genetics. 2013;45(10):1150-9.

7. Horvath S. DNA methylation age of human tissues and cell types. Genome Biol. 2013;14(10):R115.

8. Houseman EA, Accomando WP, Koestler DC, Christensen BC, Marsit CJ, Nelson HH, et al. DNA methylation arrays as surrogate measures of cell mixture distribution. BMC bioinformatics. 2012;13:86.

9. Aryee MJ, Jaffe AE, Corrada-Bravo H, Ladd-Acosta C, Feinberg AP, Hansen KD, et al. Minfi: a flexible and comprehensive Bioconductor package for the analysis of Infinium DNA methylation microarrays. Bioinformatics. 2014;30(10):1363-9.

10. Horvath S, Levine AJ. HIV-1 Infection Accelerates Age According to the Epigenetic Clock. J Infect Dis. 2015.

11. Price ME, Cotton AM, Lam LL, Farre P, Emberly E, Brown CJ, et al. Additional annotation enhances potential for biologically-relevant analysis of the Illumina Infinium HumanMethylation450 BeadChip array. Epigenetics Chromatin. 2013;6(1):4.

12. Chen YA, Lemire M, Choufani S, Butcher DT, Grafodatskaya D, Zanke BW, et al. Discovery of cross-reactive probes and polymorphic CpGs in the Illumina Infinium HumanMethylation450 microarray. Epigenetics. 2013;8(2):203-9.

13. Santpere G, Darre F, Blanco S, Alcami A, Villoslada P, Mar Alba M, et al. Genome-wide analysis of wild-type Epstein-Barr virus genomes derived from healthy individuals of the 1,000 Genomes Project. Genome biology and evolution. 2014;6(4):846-60.

14. Paisse S, Valle C, Servant F, Courtney M, Burcelin R, Amar J, et al. Comprehensive description of blood microbiome from healthy donors assessed by 16S targeted metagenomic sequencing. Transfusion. 2016.

15. Weiss S, Amir A, Hyde ER, Metcalf JL, Song SJ, Knight R. Tracking down the sources of experimental contamination in microbiome studies. Genome biology. 2014;15(12):564.

16. Turnbaugh PJ, Ley RE, Mahowald MA, Magrini V, Mardis ER, Gordon JI. An obesity-associated gut microbiome with increased capacity for energy harvest. Nature. 2006;444(7122):1027-31.

17. El Kaoutari A, Armougom F, Gordon JI, Raoult D, Henrissat B. The abundance and variety of carbohydrate-active enzymes in the human gut microbiota. Nature reviews Microbiology. 2013;11(7):497-504.

18. Lagier JC, Million M, Hugon P, Armougom F, Raoult D. Human gut microbiota: repertoire and variations. Front Cell Infect Microbiol. 2012;2:136.

19. Cayrou C, Sambe B, Armougom F, Raoult D, Drancourt M. Molecular diversity of the Planctomycetes in the human gut microbiota in France and Senegal. APMIS. 2013;121(11):1082-90.
