# Supplementary material for: Transcriptome analysis in whole blood reveals increased microbial diversity in schizophrenia
Source: Transl Psychiatry. 2018 May 10;8:96. doi: 10.1038/s41398-018-0107-9 (PMC5943399; doi:10.1038/s41398-018-0107-9)
Supplement: Supplementary file 2 — Supplementary Table 1 [file 41398_2018_107_MOESM2_ESM.pdf]

Supplementary Table 1. Data overview

A. Primary study. Whole Blood RNASeq study of 192 samples across four subject groups (Controls, ALS, BPD, SCZ)

**Disease Status**	**Control**	**SCZ**	**BPD**	**ALS**	**Total**
N	49	48	48	47	192
Number of read pairs, mean (std), millions	36.7 (6.1)	30.2 (6.1)	37.9 (4.8)	36.2 (3.4)	35.3 (6.0)
Number of mapped read pairs, mean (std), millions	26.7 (5.8)	19.7 (6.3)	29.2 (4.1)	27.7 (3.4)	25.9 (6.2)
Number of singletons* reads ,mean (std), millions	6.2 (1.5)	6.2 (1.4)	5.5 (0.9)	5.3 (1)	5.8 (1.3)
Number of unmapped pairs, mean (std), millions	3.8 (2.0)	4.3 (1.2)	3.3 (1)	3.1 (1.6)	3.6 (1.6)

*reads with one end mapped another end unmapped
** both ends of the paired-end read are unmapped

B. Positive Control. Whole blood exome sequencing from two samples

**SampleID**	Sample1	Sample2
Number of single-end reads	62,813,827	62,912,604
Number of unmapped reads	1,190,675	1,189,314

C. Negative Control. RNASeq of B-lymphoblast cell line . Samples were collected from a trio (father, mother, offspring) in duplicate, total number of sequenced samples is 6

Individual ID	Number of read pairs	Number of mapped read pairs	Number of singletons*	Number of unmapped pairs**
GM12740A	23,629,424	20,989,748	909,710	1,729,966
GM12740A	23,045,351	20,863,402	808,997	1,372,952
GM12750B	23,823,173	21,761,316	750,061	1,311,796
GM12750B	25,341,063	15,377,305	4,121,435	5,842,323
GM12751A	23,710,989	21,334,518	879,394	1,497,077
GM12751A	25,054,808	22,543,667	920,777	1,590,364

*reads with one end mapped another end unmapped

** both ends of the paired-end read are unmapped

D. Replication study. Whole Blood RNASeq study of 192 samples across two subject groups (Controls, SCZ)

Disease Status	Control	SCZ	Total
N	88	91	179
Number of read pairs, mean (std), millions	30.1 (13.2)	27.6 (11.8)	26.3 (12.0)
Number of mapped read pairs, mean (std), millions	24.1 (8.6)	19 (7.9)	20.8 (8.2)
Number of singletons* reads ,mean (std), millions	2.6 (1.2)	2.4 (1.5)	2.3 (1.3)
Number of unmapped pairs, mean (std), millions	2.6 (5.0)	4.9 (7.7)	3.2 (6.2)

*reads with one end mapped another end unmapped

** both ends of the paired-end read are unmapped
