# Supplementary material for: Transcriptome analysis in whole blood reveals increased microbial diversity in schizophrenia
Source: Transl Psychiatry. 2018 May 10;8:96. doi: 10.1038/s41398-018-0107-9 (PMC5943399; doi:10.1038/s41398-018-0107-9)
Supplement: Supplementary file 3 — Supplementary Table 2 [file 41398_2018_107_MOESM3_ESM.pdf]

**Supplementary Table 2. Similarities of blood microbiome profiles with other body sites**

| Microbial Phyla | Number of RNASeq samples | Relative abundance mean (std) | Present in more then 50% of samples in HMP | Number HMP samples taxa is detected in (Total number of sample is 499) | Reference suggesting the presence of the phylum on/in human body |
|---|---|---|---|---|---|
| Fusobacterium | 1 | 0.041 (0.000) | oral, stool | 309 | Human Microbiome Project, 2012 |
| Elusimicrobia | 3 | 0.026 (0.012) | N/A | 0 | N/A |
| Acidobacteria | 3 | 0.032 (0.011) | N/A | 3 | gut, Andersson, Anders F., et al. 2013 |
| Tenericutes | 3 | 0.051 (0.030) | no | 14 | Human Microbiome Project, 2012 |
| Deinococcus-Thermus | 4 | 0.025 (0.012) | N/A | 0 | gut, Lagier, Jean-Christophe, et al. 2012 |
| Synergistetes | 5 | 0.036 (0.025) | N/A | 0 | N/A |
| Aquificae | 5 | 0.057 (0.036) | N/A | 2 | gut, Lagier, Jean-Christophe, et al. 2012 |
| Nitrospirae | 6 | 0.039 (0.014) | N/A | 0 | N/A |
| Spirochaetes | 6 | 0.037 (0.020) | oral | 143 | Human Microbiome Project, 2012 |
| Chlamydiae | 8 | 0.038 (0.023) | oral, stool | 249 | Human Microbiome Project, 2012 |
| Chloroflexi | 9 | 0.045 (0.025) | no | 35 | Human Microbiome Project, 2012 |
| Verrucomicrobia | 11 | 0.048 (0.033) | no | 65 | Human Microbiome Project, 2012 |
| Deferribacteraceae | 14 | 0.044 (0.014) | oral, stool | 248 | Human Microbiome Project, 2012 |
| Thaumarchaeota | 18 | 0.041 (0.015) | N/A | 0 | skin, Probst, Alexander J. 2013 |
| Bacteroidetes | 25 | 0.067 (0.047) | oral, stool, skin | 375 | Human Microbiome Project, 2012 |
| Planctomycetes | 30 | 0.060 (0.029) | N/A | 0 | gut, Cayrou, Caroline, et al.2013 |
| Thermotogae | 35 | 0.064 (0.036) | N/A | 0 | gut, Kaoutari et al., 2013 |

| | | | | | |
|---|---|---|---|---|---|
| Euryarchaeota | 48 | 0.072 (0.035) | no | 77 | Human Microbiome Project, 2012 |
| Crenarchaeota | 55 | 0.057 (0.025) | N/A | 0 | gut, Turnbaugh et al., 2006 |
| Actinobacteria | 56 | 0.057 (0.047) | airways,oral, stool,skin | 438 | Human Microbiome Project, 2012 |
| Cyanobacteria* | 94 | 0.110 (0.089) | no | 14 | Human Microbiome Project, 2012 |
| Firmicutes* | 140 | 0.149 (0.109) | airways,oral, stool,skin | 450 | Human Microbiome Project, 2012 |
| Proteobacteria* | 190 | 0.734 (0.183) | airways,oral, stool,skin | 399 | Human Microbiome Project, 2012 |

*present in 1/4 of the samples of each subject group
